# Aromatic Copolyamides with Anthrazoline Units in the Backbone: Synthesis, Characterization, Pervaporation Application

**DOI:** 10.3390/polym8100362

**Published:** 2016-10-17

**Authors:** Galina A. Polotskaya, Alexandra Yu. Pulyalinа, Mikhail Ya. Goikhman, Irina V. Podeshvo, Irina A. Valieva, Alexander M. Toikka

**Affiliations:** 1 Department of Chemical Thermodynamics and Kinetics, Saint Petersburg State University, Saint Petersburg 198504, Russia; a.pulyalina@spbu.ru (A.Y.P.); a.toikka@spbu.ru (A.M.T.); 2 Institute of Macromolecular Compounds, Russian Academy of Sciences, Saint Petersburg 199004, Russia; goikhman@hq.macro.ru (M.Y.G.); podeshvo@hq.macro.ru (I.V.P.); valieva@hq.macro.ru (I.A.V.)

**Keywords:** copolyamides with anthrazoline units, membranes, sorption, pervaporation, methanol–toluene mixture

## Abstract

Copolyamides with anthrazoline units in the backbone (coPA) were synthesized and dense nonporous films were prepared by solvent evaporation. Glass transition temperature, density, and fractional free volume were determined for the dense nonporous films composed of polyamide and two of its copolymers containing 20 and 30 mol % anthrazoline units in the backbone. Transport properties of the polymer films were estimated by sorption and pervaporation tests toward methanol, toluene, and their mixtures. An increase in anthrazoline fragments content leads to an increasing degree of methanol sorption but to a decreasing degree of toluene sorption. Pervaporation of a methanol–toluene mixture was studied over a wide range of feed concentration (10–90 wt % methanol). Maximal separation factor was observed for coPA-20 containing 20 mol % fragments with anthrazoline units; maximal total flux was observed for coPA-30 with the highest fractional free volume.

## 1. Introduction

Polymers of heteroaromatic structures exhibit high thermal stability, excellent mechanical strength, high resistance to organic solvents, and other valuable properties that find various applications in a broad range of industries, such as aerospace engineering, mechanical engineering, electrical engineering, microelectronics, etc. [1,2]. Structural ordering, fixed free volume, and physicochemical properties of polymers make them especially useful as efficient membrane materials for the separation of liquid and gas mixtures [3,4,5,6,7,8,9]. The chemical structure of the monomer unit is the dominant factor determining the physical and chemical properties of polyheteroarylenes, including membrane transport parameters [10,11,12,13]. It should be noted that the transport properties are more sensitive to changes in the chemical structure as compared to the majority of physical characteristics [14,15].

Pervaporation is a very promising membrane technology of liquid mixture separation that does not require additional chemicals and is more energy saving, environmentally safe, and a clean technology as compared to distillation [16]. In pervaporation, the feed liquid mixture is placed in contact with the upstream side of the membrane, while the vapor permeate is removed from the downstream side under vacuum application [17]. The efficiency of the pervaporation depends on the type of chosen membrane [18,19].

Polymers with anthrazoline units in the backbone have attracted attention very recently. It has been found that these polymers combine the generic advantages of polymers (mechanical strength, heat resistance, and processability) with a number of peculiar characteristics of anthrazoline (optical properties, semiconductor and others) [20,21,22]. In [23] it was demonstrated that polyanthrazolines can be considered as promising electronic and optoelectronic materials. At the same time, application of polymers with anthrazoline units in membrane technologies has not been studied so far.

In the present work, the effect of anthrazoline fragments inclusion into aromatic polyamide was estimated for dense nonporous films prepared from polyamide (PA) and two copolyamides (coPA) containing 20 and 30 mol % anthrazoline units in the backbone. Some physical properties and thermogravimetric analysis of these objects were studied. Mass transfer of methanol, toluene, and their mixtures through the above mentioned three films was investigated by sorption and pervaporation techniques.

The problem of methanol and toluene separation is very important for the oil refining industry. It is known that toluene can be obtained in the process of catalytic reforming of gasoline oil fractions [24]. To separate toluene from the gasoline, methanol is used for two purposes, as a leach for extraction and as an entrainer for distillation. Finally, to obtain pure toluene, its separation from the residual methanol is required, especially as they form an azeotrope at 69 wt % methanol and 31 wt % toluene. A traditional way to break the azeotrope is the distillation with addition of entrainers [25]. To separate a methanol–toluene mixture, membranes based on cellulose derivatives, polyimide, polyamide, polyphenylene oxide, polysiloxane, chitosane etc. have been used [26,27,28,29,30,31,32,33,34,35]. Comparative analysis of these membranes and novel coPA membranes for pervaporation of a methanol–toluene mixture in composition close to the azeotropic point was among the objectives of the present work.

## 2. Materials and Methods

### 2.1. Materials

Methanol and toluene of chemically pure (CP) grade were purchased from Vekton (Saint Petersburg, Russia) and were used as received. *N*-methylpyrrolidone (NMP) and propylene oxide were purchased from Aldrich (St. Louis, MO, USA) and were used as received. The compound 4,4′-diaminodiphenyl ether was purified by vacuum distillation, mp = 190 °C.

Copolyamides were synthesized via low-temperature polycondensation using dichloroanhydride and two diamines. The dichloroanhydride of terephthaloyl-bis(3-methoxy-4-oxybenzoic) acid was synthesized according to the procedure described in [36]. Diamine (**1**) {2,8-bis(4-aminophenyl)pyrido[3,2-g]quinoline-4,6-dicarboxylic acid} was synthesized using pyrrolo[3,2-f]indol-2,3,5,6(1H,7H)-tetraone [37] and *p*-aminoacetophenone.

### 2.2. Polymer Synthesis

A method for producing a coPA-20 as well as the scheme of the reaction is given below.

**Figure 1:**
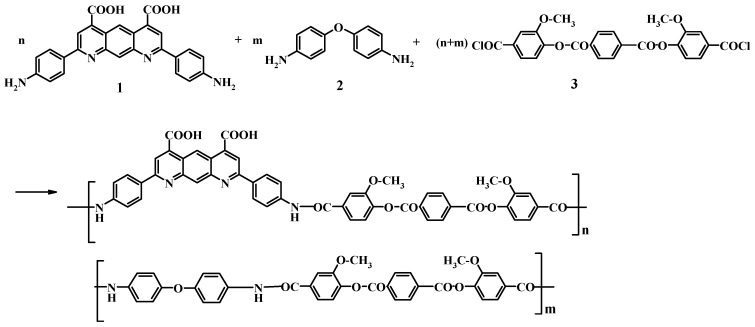
The scheme of copolyamide (coPA) synthesis.

A flask equipped with a stirrer was filled with 0.2 mmol of diamine (**1**), 0.8 mmol of diamine (**2**) and 5 mL NMP, solution and cooled to −15 °C. Then the dichloroanhydride of terephthaloyl-bis(3-methoxy-4-oxybenzoic) acid (**3**) was added. After the suspension had been stirred for 30 min at –15 °C, 0.1 mL of propylene oxide was added, and the cooling was stopped. The suspension was stirred at room temperature for 2 h to form a viscous, transparent solution, then the stirring was continued for 3 h. The scheme of coPA synthesis is shown on Figure 1.

The coPA-20 solution was filtered and used for the film preparation without any further purification.

Polyamide (PA) and coPA-30 were synthesized by a similar procedure. A specific amount of anthrazoline units in the copolyamide backbone (20 and 30 mol %) was chosen in order to avoid gelation in the polymer solutions, which can be used for preparation of films with good mechanical properties.

### 2.3. Film Preparation

Dense polymer films were obtained by casting polymer solutions in NMP on a glass plate, followed by evaporation of the solvent at 100 °С in air. Films fixed on the glass plate were dried to a constant weight at 50 °С under vacuum for 5 days. The polymer film thicknesses measured by the digital micrometer (Etalon, Moscow, Russia) were around 30–40 µm with an error ±1 µm.

### 2.4. Thermogravimetric Analysis (TGA)

TGA was carried out using samples of ~10–14 mg. They were put in a platinum crucible; heating speed was 10 °C/min in a nitrogen atmosphere. The TG 209 F3 Iris thermo-microbalance (Netzsch, Selb, Germany) was used for the analysis.

### 2.5. Scanning Electron Microscopy

Membrane morphology was studied by scanning electron microscope SEM Zeiss SUPRA 55VP (Oberkochen, Germany). Before the test the sample surface was covered by a gold layer via cathode sputtering using the Quorum 150 (East Sussex, UK) installation.

### 2.6. Physical Properties

The glass transition temperature (*T*_g_, °C) was determined using a differential scanning calorimeter DSC 204 F1 (Netzsch, Selb, Germany). The analysis was carried out under an inert atmosphere with 4–5 mg samples at a scan rate 10 °C/min from −20 to 300 °C.

The film density (*ρ*, g/cm^3^) was estimated using the flotation method with a laboratory-made measurement unit [12]. Samples of 0.05–0.10 g were used; the error of measurements was ±0.002 g/сm^3^.

Fractional free volume (*FFV*) of the polymer film was calculated using Bondi’s method by the following equation [38]:

*FFV* = (*V*_0_ − 1.3·*V_w_*)/*V*_0_ (1)

where *V*_0_ = 1/*ρ* is the polymer specific volume and *V_w_* = *N_A_*·Σ*V_i_* is the van der Waals volume of the repeated unit of polymer; *V_i_* is contribution of each atom to van der Waals volume and its magnitude is given in [39].

Hildebrand solubility parameter {*δ*, (J/сm^3^)^0.5^} of the repeated unit for the homopolymer was determined as [39,40]:

(2) $$\delta ={\left(\frac{\Delta E_{w}}{V_{w}}\right)}^{1/2}={\left(\frac{\sum_{i}\Delta E_{i}}{V_{w}}\right)}^{1/2}$$

where Δ*E_i_* is the contribution of each atom and intermolecular interaction to the cohesive energy ∆*E_w_*, which was calculated as an additive magnitude using the table data [41].

The Hildebrand solubility parameter (*δ*) of the repeated unit for copolymer was calculated according to works [39,40].

(3) $$\delta ={\left(\frac{\alpha_{1}{\left(\sum_{i}\Delta E_{i}\right)}_{1}+\left(1-\alpha_{1}\right){\left(\sum_{i}\Delta E_{i}\right)}_{2}}{N_{A}\left[\alpha_{1}{\left(\sum_{i}\Delta V_{i}\right)}_{1}+\left(1-\alpha_{1}\right){\left(\sum_{i}\Delta V_{i}\right)}_{2}\right]}\right)}^{1/2}$$

where α_1_ is the mole fraction of Component 1 in the copolymer, (ΣΔ*E_i_*)_1_ and (ΣΔ*E_i_*)_2_ are the cohesive energy of Components 1 and 2 in the copolymer, (Σ*V_i_*)_1_ and (Σ*V_i_*)_2_ are the van der Waals volume of Components 1 and 2 in the copolymer.

### 2.7. Sorption Experiment

The sorption experiment was performed by immersing of 0.10–0.12 g sample (additionally dried at 50 °С under vacuum for 7 days) into liquid methanol, toluene, or their mixtures under atmospheric pressure at 20 °С. The weight change was determined gravimetrically within the error ±10^−4^ g. The experiment was continued until equilibrium was attained (4 days for methanol and around 2 weeks for toluene and mixtures). After completion of sorption experiments, the solvent desorption was carried out by exposing the samples at 20 °С in the controlled environment of the desiccator containing a molecular sieve absorber. The sorption experiments for all samples were repeated three times with similar results.

The kinetic curves of sorption were plotted. The equilibrium sorption degree (*S*, %) was calculated from the weight difference between the wet membranes (*m*_wet_, g) at equilibrium and the weight of dry sample (*m*, g) as shown in the equation [41,42]:

(4) $$S=\frac{m_{\mathrm{wet}}-m}{m}$$

The thermodynamic polymer-solvent interaction parameter ($\chi_{1}$), expressing affinity between the polymer and the penetrant, was defined using the equation of the Flory–Huggins theory [43]:

(5) $$\ln a_{1}=\;\left[\ln\left(1-\varphi_{2}\right)+\varphi_{2}+\chi_{1}\varphi_{2}^{2}\right]$$

where $a_{1}$ is the penetrant activity, $\varphi_{2}$ is the penetrant volume fraction in a swollen polymer sample expressed as:

(6) $$\varphi_{2}=\frac{1}{1+\frac{\rho_{2}}{\rho_{1}}\Delta s}$$

where $\rho_{1}$ and $\rho_{2}$ are density of penetrant and polymer, respectively; ∆*s* is the amount of penetrant sorbed by polymer (g/g).

For a liquid penetrant $a_{1}$ = 1, and $\chi_{1}$ was calculated by the following equation:

(7) $$\chi_{1}=\frac{-\left[\ln\left(1-\varphi_{2}\right)+\varphi_{2}\right]}{\varphi_{2}^{2}}$$

### 2.8. Pervaporation

The pervaporation experiment was carried out on the laboratory cells with an effective membrane area of 14.8 сm^2^ at 20, 35, and 50 °C with stirring [44]. Downstream pressure below 10^−2^ mm Hg was maintained. The feed was a methanol–toluene mixture. The permeate was collected into a liquid nitrogen cooled trap, weighed, and analyzed. The composition of feed mixture and permeate was determined using a gas chromatograph (Chromatec–Crystal 5000.2, Chromatec Company, Yoshkar-Ola, Russia) with a thermal conductivity detector with the error ±10^−3^ wt %.

From the pervaporation experiments, the total permeation flux and separation factor were calculated [45,46]. The separation factor (α*_M_*_ethanol/Toluene_) was determined using the following equations:

(8) $$\alpha_{ij}=(Y_{i}/Y_{j})/(X_{i}/X_{j})$$

where subscripts *i* and *j* refer to methanol and toluene, respectively; *Y* and *X* are the weight fractions of the component in the permeate and feed, respectively.

Total permeation flux (*J*, g/m^2^h) was determined as the amount of liquid penetrated through the membrane area per time unit:

(9) $$J=\frac{Q}{S\cdot t}$$

where *Q* is the total weight of the permeate (g) collected at time *t* (h), *S* is the effective surface area of the membrane (m^2^).

To compare membranes with different thickness (*l*, μm) varying from 30 to 40 µm, the value of normalized flux (*J_n_*) was used. *J_n_* is the flux through the membrane of 20 µm thickness calculated as:

(10) $$J_{n}=\frac{J\cdot l}{20}$$

For the evaluation of the intrinsic properties of the penetrant-membrane system, permeance and selectivity were calculated [47]. Membrane permeance (*P_i_/l*) is a component of flux normalized for driving force and was found by using the following equation:

(11) $$\frac{P_{i}}{l}=\frac{j_{i}}{p_{i0}-p_{il}}$$

where *j_i_* is a molar flux of component *i*, cm^3^(STP)/cm^2^·s, and $p_{i0}$ and $p_{il}$ (cm Hg) are the partial pressures of component *i* on both sides of the membrane (0 stands for the surface on the feed side and *l* stands for the surface on the permeate side). Permeance was calculated in GPU (1 GPU = 1 × 10^−6^ (cm^3^(STP)/cm^2^·s·cm Hg).

Membrane selectivity (β*_M_*_ethanol-Toluene_) was defined as the ratio of the permeances:

(12) $$\beta_{ij}=\frac{P_{i}/l}{P_{j}/l}$$

where subscripts *i* and *j* refer to methanol and toluene, respectively.

## 3. Results and Discussion

### 3.1. Physical and Thermal Properties

Polycondensation was used to synthesize polyamide (PA) and two of its copolymers: coPA-20 and coPA-30, containing 20 and 30 mol % anthrazoline units in the backbone, respectively. The molecular weights of coPA-20 and coPA-30 were determined by gel permeation chromatography (Agilent 1260 Infinity II LC, Santa Clara, CA, USA) and were equal to 43 and 41 kD, respectively. The composition of the copolymers obtained by polycondensation in the case of a polymer with a high molecular weight usually corresponds to the initial ratio of monomers. FTIR spectra of coPA films were recorded in the range of 450–4000 cm^−1^ on a Shimadzu FTIR-8400S spectrophotometer. In the spectra absorption bands at 1615 cm^−1^ (C–N) and 1590 cm^−1^ (C–C) were observed, which were assigned to the anthrazoline units in the coPA structure.

**Figure 2:**
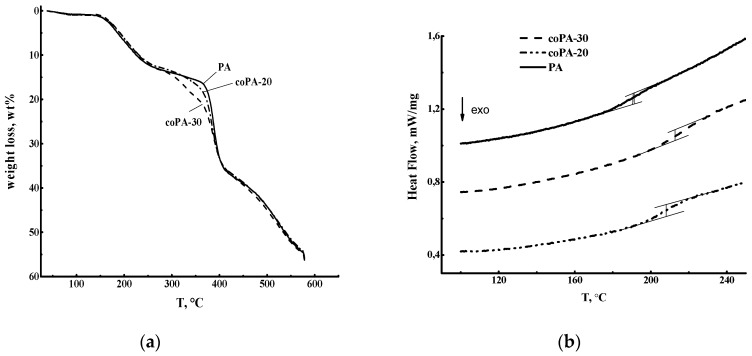
(**a**) Thermogravimetric (TG) and (**b**) differential scanning calorimetry (DSC) curves of PA, coPA-20, and coPA-30.

TGA and DSC were used to determine the thermal stability of polyamide and its copolymers with 20 and 30 mol % fragments with anthrazoline units (Figure 2). TGA revealed three temperature ranges where appreciable weight loss of the samples occurs (Figure 2a). The first range of the weight loss about 1 wt % is observed up to 100 °C; it is associated with the removal of water adsorbed from atmospheric moisture. With further heating, the speed of weight loss increases sharply: in a fairly narrow range from 120 to 280 °C all samples lose about 12% of their weight. At this stage, the residual solvent NMP is removed and destruction of the amide groups of the polymer chain begins. Finally, in the range from 300 to 420 °C there is another stage of intensive sample destruction and weight loss up to 22–24 wt % is observed. This stage involves thermal destruction of the aromatic groups (mainly anthrazoline) of the polymer chain, and the speed of polymer destruction increases with the content of anthrazoline units.

The second heating cycle of DSC was used to determine the glass transition temperature of the polymers under study (Figure 2b).

Рhysical properties such as the glass transition temperature (*T*_g_), density, fractional free volume (*FFV*), and the solubility parameters were determined for the dense nonporous films composed of polyamide and its copolymers with anthrazoline units in the backbone. Table 1 shows that the *T*_g_ increases with an increase in anthrazoline units content in the copolymer. The density of PA films is larger than those of coPA. The values of fractional free volume calculated according to the Askadskii method [39] increases with the introduction of anthrazoline units. This fact points to the relocation of free volume and structure transformation in the coPA films.

Solubility parameter data presented in Table 1 allows prediction of the solubility of different organic liquids in our polymer films. According to the solubility theory [40], the less the difference in solubility parameters of polymer and penetrant |∆δ|, the better is the solubility of the penetrant in the polymer. The liquids under study were methanol and toluene that exhibit solubility parameters equal to 29.7 and 18.2 (J/cm^3^)^0.5^, respectively. It was found that methanol solubility should be preferential compared to toluene.

### 3.2. Films Structure

**Figure 3:**
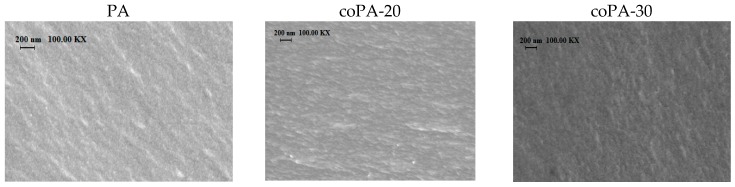
Scanning electron microscopy (SEM) micrographs of cross-section of PA, coPA-20, and coPA-30 films.

Scanning electron microscopy was used to study the morphology of dense films (Figure 3). The cross-sections of PA, coPA-20, and coPA-30 films are characterized by a homogeneous uniform structure containing small elements of a supramolecular structure (≤50 nm). Micrographs of the cross-section prove the lack of defects in all films under study.

### 3.3. Sorption Study

**Figure 4:**
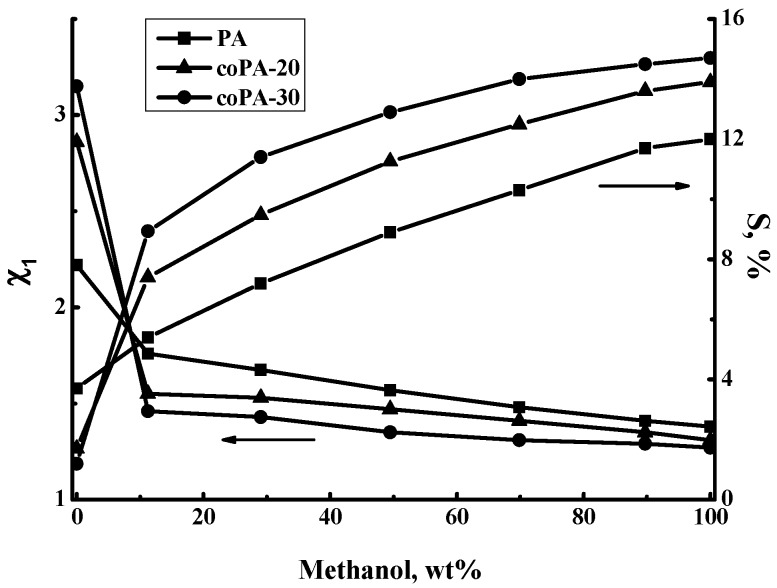
Dependence of equilibrium sorption degree and interaction parameter $\chi_{1}$ on methanol concentration in methanol–toluene mixtures, 20 °C.

Sorption experiments were carried out by immersion of polymer film samples into the individual liquids (methanol and toluene) or into their mixtures to determine data on the equilibrium sorption degree and the Flory–Huggins interaction parameter ($\chi_{1}$) of the liquids under study as well as the polymer films. Figure 4 shows the dependence of the equilibrium sorption degree and interaction parameter $\chi_{1}$ on the methanol concentration in methanol–toluene mixtures for PA, coPA-20, and coPA-30 films.

The PA film exhibits the highest degree of toluene sorption. The increase of anthrazoline units content leads to a decreasing degree of toluene sorption. With the growth of the methanol concentration in the methanol-toluene mixture, the sorption degree increases for all polymer films. Figure 4 shows the lower sorption activity of PA film with respect to the methanol‒toluene mixture while inclusion of fragments with anthrazoline units in the film leads to an increase in the sorption degree. The sorption degree of pure methanol is significantly higher than that of toluene. Copolymers with anthrazoline units demonstrate a higher degree of methanol sorption as compared to PA film.

The mass transfer through the polymer film depends substantially on the affinity of the liquid to the polymer. Quantitatively, the liquid–film interaction can be expressed in terms of the Flory–Huggins parameter, $\chi_{1}$ [43]. Usually, a stronger interaction between the polymer and the liquid would result in a smaller magnitude of $\chi_{1}$ because the amount of liquid inside the polymer is higher. In contrast, lower affinity between the polymer and the liquid would result in a higher $\chi_{1}$ magnitude [43,45]. As it can be seen from Figure 4, the lower magnitude of the Flory–Huggins parameter $\chi_{1}$ is attributed to mixtures with high methanol concentration and the lowest magnitude is achieved for pure methanol which interacts with the films better than toluene. Parameter $\chi_{1}$ for the mixtures with high methanol concentration and pure methanol decreases from PA to coPA-30; this means that interaction between polymer films and liquid increases. While parameter $\chi_{1}$ for toluene increases from PA to coPA-30, the interaction between the polymers and liquid decreases. Higher affinity to the methanol and methanol-toluene mixture (lower $\chi_{1}$) was found for copolymer with the higher content of fragments with anthrazoline units as compared with PA.

Sorption–diffusion parameters have an effect on the transport properties in pervaporation. It should be noted that thermodynamic analysis of sorption in terms of pervaporation performance has been successfully made on the basis of the Flory‒Huggins theory in some experimental and theoretical works [48,49,50]. Such an approach allows an explanation for the pervaporation processes based on the sorption–diffusion mechanism.

### 3.4. Pervaporation of Methanol–Toluene Mixture

**Figure 5:**
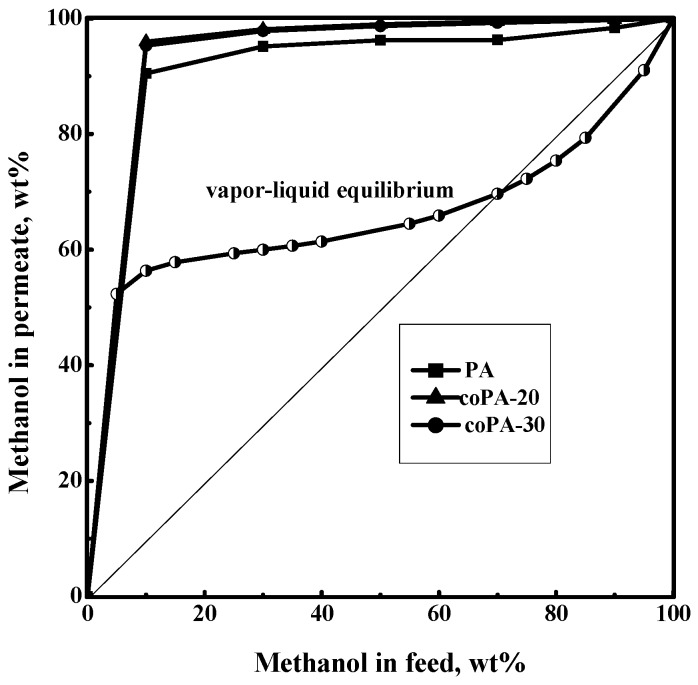
Dependence of methanol concentration in the permeate on methanol concentration in the feed for the pervaporation of a methanol–toluene mixture. Vapor–liquid equilibrium curve of methanol and toluene mixture, 760 mm Hg.

All polymer films were studied in the pervaporation of a methanol–toluene mixture over the entire range of concentration. Figure 5 shows the dependence of methanol concentration in the permeate on methanol concentration in the feed for the pervaporation of a methanol–toluene mixture at 20 °C using three types of films. To compare results of the pervaporation tests, Figure 5 shows also a vapor–liquid equilibrium curve for the methanol–toluene system at 760 mm Hg [51]. The run of the vapor–liquid equilibrium curve differs essentially from the permeate–feed concentration curves in the pervaporation of the methanol–toluene mixture. The permeate is considerably enriched with alcohol and contains more than 95 wt % methanol for coPA films.

**Figure 6:**
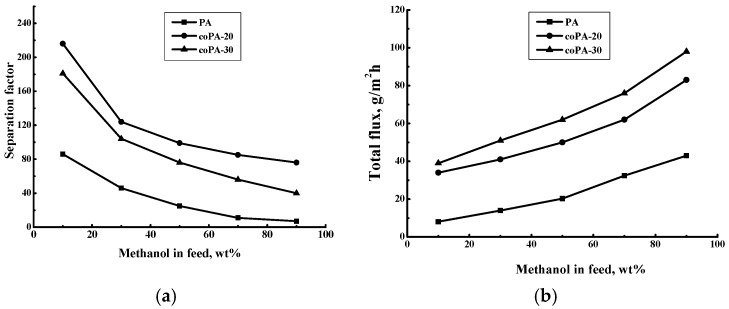
Dependence of (**a**) separation factor *α*_Methanol/Toluene_ and (**b**) total flux on methanol concentration in the feed for the pervaporation of a methanol–toluene mixture, 20 °C.

Figure 6 shows dependences of the separation factor α_Methanol-Toluene_ and the total flux on the methanol concentration in the feed. For all films the separation factor decreases with increase of methanol concentration in the feed. The maximal separation factor was observed for coPA-20 film. The separation factor of coPA-30 film is higher than that of PA film. The increase of methanol concentration facilitates film swelling and growth of transport canals. Such a trend leads to facilitating the permeation of large toluene molecules and decreasing the separation factor of the films.

The total flux through 20 µm thick membranes increases with the growth of the more permeable component (methanol) concentration in the feed. Maximal total flux was observed for the coPA-30 film. The total flux position of coPA-20 is lower than that of coPA-30. The PA film is characterized by the lowest separation factor (methanol–toluene) and total flux as compared to both copolymer films over the entire range of feed concentration.

The highest separation factor in the pervaporation of the methanol–toluene mixture was observed for the coPA-20 film. Maximal total flux was obtained for the coPA-30 film. The opposite trend is determined by the higher free volume of coPA-30 than that of coPA-20. This fact promotes the easier transport of both liquids through coPA-30 which provides the larger flux and lower separation factor of coPA-30 compared to coPA-20.

**Figure 7:**
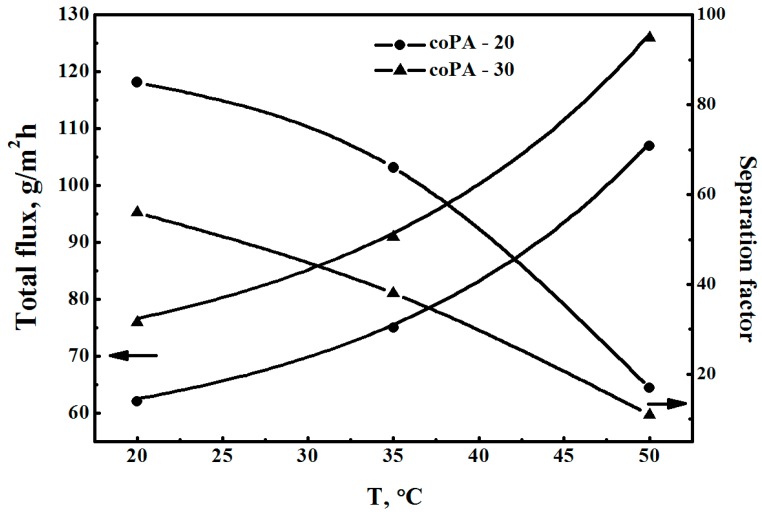
Dependence of total flux and separation factor α_Methanol/Toluene_ on temperature for the pervaporation of azeotropic methanol–toluene (70:30 wt %) mixture through coPA films, 20 °С.

Hence, coPA films exhibit better total flux and separation factor than that of homo-PA in the pervaporation of a methanol–toluene mixture. Figure 7 shows temperature dependences of the total flux and the separation factor of coPA films in the pervaporation of azeotropic methanol–toluene (70:30 wt %) mixture. It is natural that an increase in the temperature leads to an increase of the total flux and to a decrease of the separation factor [17,46]. A satisfactory performance of these films in the pervaporation of azeotropic methanol–toluene mixture at 20, 35, and 50 °C was revealed.

**Figure 8:**
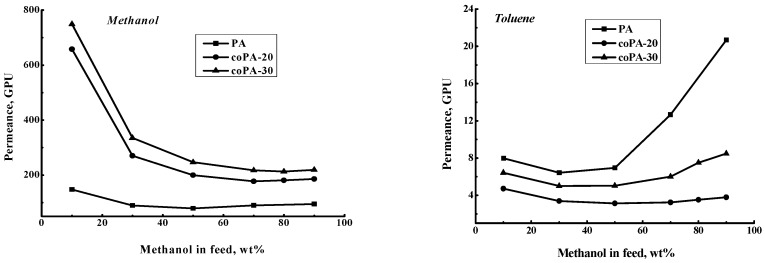
Dependence of methanol and toluene permeance on methanol concentration in the feed, 20 °С.

Intrinsic properties of the penetrant-film system were evaluated by calculation of the permeance and selectivity (β_Methanol-Toluene_) [47]. Figure 8 demonstrates the dependence of methanol and toluene permeance on the methanol concentration in the feed. The methanol permeance is high at low methanol concentration in the feed due to the high affinity of films to methanol. The toluene permeance, on the contrary, becomes better at high methanol concentration in the feed, when the film is more swollen and the penetration of large toluene molecules is facilitated. It was shown that the introduction of fragments with anthrazoline units in the backbone leads to maximal methanol permeance for coPA-30 and maximal toluene permeance for coPA-30. This fact is reflected in data of the pervaporation experiments for the total flux and separation factor (Figure 6).

**Figure 9:**
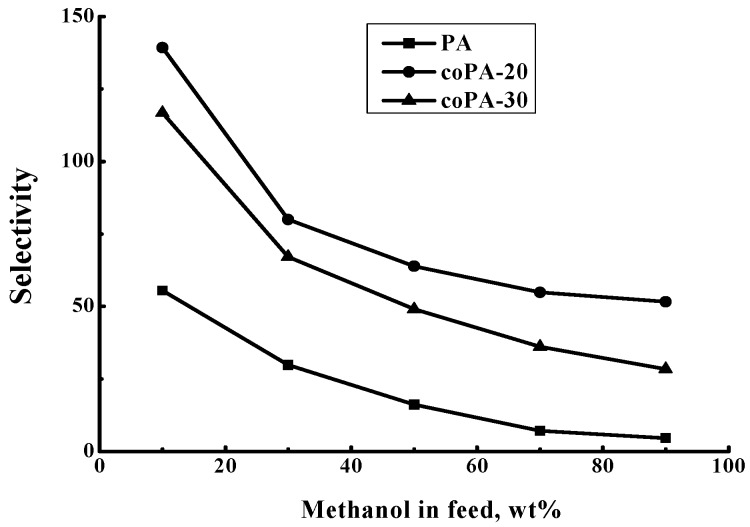
Dependence of selectivity β_Methanol-Toluene_ on methanol concentration in the feed for the pervaporation of a methanol–toluene mixture, 20 °С.

Figure 9 demonstrates dependence of selectivity β_Methanol__-Toluene_ on methanol concentration in the feed. In general, when the driving force effect is removed, the run of selectivity curves (Figure 9) is similar to that of the separation factor (Figure 6). However, the selectivity magnitudes are lower than the magnitudes of the separation factor. The higher value of the separation factor is related to a much higher volatility of methanol compared to toluene which facilitates separation of the methanol-toluene mixture.

### 3.5. Comparison of Pervaporation Properties with Literature Data

The mass transfer of a methanol–toluene mixture with a composition close to the azeotropic point is of special interest. The transport properties of the copolyamides synthesized in this work were compared with literature data for the case of the pervaporation separation of a methanol–toluene mixture (70:30 wt %). Table 2 shows data on the total flux and methanol concentration in permeate obtained for different polymers and their compositions from a number of papers [25,26,27,28,29,30,31,32,33,34,35]. The last two columns show the methanol permeance and selectivity of the films calculated from the reported data with Equations (11) and (12). Both the methanol permeances and selectivity of coPA-20 and coPA-30 are at a moderate level compared with that of the previously published data. It should be noted that there is the possibility of improving the performance of coPA membranes, probably by development of composite membranes or other modes and this will be undertaken in the future.

## 4. Conclusions

The effect of anthrazoline fragments inclusion in aromatic polyamide was estimated by comparing dense nonporous films prepared from aromatic polyamide and two copolyamides containing 20 and 30 mol % anthrazoline units in the backbone. It was found that the glass transition temperature, the density of films, and the magnitudes of the fractional free volume increase with the introduction of anthrazoline units which points to structure transformation in the polymer films. TGA showed that weight loss of basic PA and coPA are similar up to 300 °C and only in the range 300–420 °C does the speed of polymer destruction increase with the concentration of anthrazoline fragments.

Sorption studies show that the equilibrium degree of methanol sorption is significantly higher than that of toluene for all polymer films. The increase of anthrazoline fragments content leads to an increasing degree of methanol sorption but to a decreasing degree of toluene sorption. The Flory–Huggins interaction parameters calculated by using data of sorption experiments in methanol–toluene mixtures indicate a higher affinity of copolymers with the highest content of fragments with anthrazoline units as compared with PA.

Transport properties were studied in pervaporation of a methanol–toluene mixture over the entire range of feed concentration. It was found than basic PA is characterized by the lowest performance in separating methanol from toluene as compared to both copolymers. The maximal separation factor was observed for coPA-20 containing 20% fragments with anthrazoline units in the backbone; maximal total flux was observed for coPA-30. To evaluate the intrinsic properties of the penetrant-polymer system, permeance and selectivity were calculated. The permeance of both penetrants, methanol and toluene, goes up with increasing concentration of anthrazoline fragments.

Comparison of the transport properties of the present coPA films with literature data was made for the pervaporation of methanol–toluene (70:30 wt %) mixture with a composition close to the azeotropic point. Both the methanol permeances and magnitudes of selectivity of coPA-20 and coPA-30 are at a moderate level, in comparison with the results of previous studies by other authors.

## Figures and Tables

**Table 1:** Physical properties of polymer films.

Film	Glass transition temperature, °C	Density, g/сm^3^	Solubility parameter *δ*, (J/сm^3^)^0.5^	Fractional free volume
PA	184	1.307	24.84	0.342
coPA-20	204	1.313	24.92	0.355
coPA-30	211	1.319	24.96	0.361

**Table 2:** Comparison of transport properties of the present coPA films with literature data on pervaporation of methanol–toluene (70:30 wt %) mixture.

Polymer	*T*, °C	Total flux, g/m^2^h	Methanol in permeate, wt %	Permeance (*P/l*)_Methanol_, GPU	Selectivity (β_Methanol-Toluene_)	References
coPA-20	50	107.0	97.5	301	11	Present work
coPA-30	50	126.0	96.3	316	7.0	
Polyamide/MMT (3%)	50	112	99.2	78	11	[26]
Polyimide	50	16.0	92.1	9.9	1.0	[27]
Polyimide/polyanyline	50	12.5	94.9	8.0	1.6	[27]
Poly(phenyleneoxide)	30	300	98.6	502	19	[28]
Cellulose acetate	30	1400	92.1	2188	3.2	[29,30]
Cellulose triacetate	30	370	92.1	578	3.2	[29,30]
Cellulose acetate/Cellulose butyrate	30	500	92.1	781	3.2	[29]
Polyphthalamide/poly(vinylalcohol)	30	250	99.6	422	68	[31]
Poly(vinylalcohol)/(Hydroxy(ethyl) methacrylate + aceticanhydride)	30	1100	98.6	1840	19	[32]
Chitosan	35	1000	99.4	1318	45	[33]
Chitosan/aceticanhydride (4%)	35	1000	99.3	1932	57	
Copolymer of perfluoro-2,2-dimethyl-1,1,3-dioxole and tetrafluoroethylene (25% toluene)	50	1.7	95.2	96	15	[34]
Interpenetrating network of vinylterminated poly(dimethylsiloxane) and aromatic polyimide (90:10)	30	52	92.3	2839	9.3	[35]
